# New Features on Mechanical Behavior of Polymeric Materials

**DOI:** 10.3390/polym18020266

**Published:** 2026-01-19

**Authors:** Emilia P. Collar, Jesús-María García-Martínez

**Affiliations:** Polymer Engineering Group (GIP), Polymer Science and Technology Institute (ICTP), Spanish National Research Council (CSIC), C/Juan de la Cierva, 3, 28006 Madrid, Spain

This Special Issue, devoted to new features on “Mechanical Behavior of Polymeric Materials”, includes many exciting works related to this frontrunner polymer Research and Development area. The topic’s fundamentals underscore their significance and address a dynamic, rapidly advancing area of polymer science. Specifically, it examines the diverse factors that influence the properties of polymer-based materials. It is essential to provide readers with foundational information on the concept of failure in polymer-based materials, especially as it relates to their mechanical properties. From a fundamental perspective, a fracture in a material occurs when the forces binding its constituent atoms are overcome. However, the specific ways in which these atoms are bonded in molecules and the resulting diversity of supramolecular aggregates lead to complex questions and contribute to the heterogeneous nature of these materials [1,2]. In evaluating material strength, it has been well established since the early studies by Griffith [3] that fracture consistently initiates in the weakest region of a material. However, the pursuit of empirical and semi-empirical approaches, grounded in the results of rigorously designed mechanical testing procedures, remains an open research question, particularly when these procedures are limited to material or part performance. These considerations are particularly significant for heterogeneous materials composed entirely or partially of organic polymers. Since the early stages of materials research [4,5,6], it has been established that the stress–strain relationship in such materials is nonlinear, as it is strongly influenced by time- and temperature-dependent effects on the applied external force, as well as by morphological changes within the material’s bulk resulting from environmental conditions [7,8,9]. This underscores the continued importance of research in this field to fulfill key scientific requirements and ensure reproducible, reliable results. The development of inter-laboratory protocols and international standardized procedures is crucial for providing robust estimates of mechanical parameters [10,11,12,13,14,15]. These values should be suitable for incorporation into comprehensive databases to support polymeric part and material design software [16], enabling accurate forecasting of the mechanical behavior of polymeric materials once they reach the solid-state following processing steps that determine the resulting morphologies of the organic fractions in these materials [17,18]. Recent findings demonstrate that the interaction between mechanical energy and matter varies significantly across different environments and conditions. This, combined with rigorous analysis of material responses, remains essential for developing mathematical models to optimize material design within a comprehensive sustainability framework, ultimately aiming to prevent catastrophic failure scenarios.

To mention that a substantial number of manuscripts (15 articles) were submitted for consideration in this Special Issue; however, only a select group (eight documents) was published after the rigorous review process of *Polymers*, yielding a 47% acceptance rate. The articles compiled in this volume are fully aligned with the research philosophies outlined above. It is noteworthy to note that the purpose of this Editorial is not to discuss each article in detail, but rather to encourage readers to engage with them thoroughly. Therefore, since this editorial aims not to elaborate on each text but to encourage the reader to browse them in depth, these contributions have been briefly described below. For such purposes, a short note on each one has been reported to awaken interest in each of the contributions to this exciting Special Issue of *Polymers*, rather than providing an exhaustive description of each of them.

In this way, the article by Sun et al. [19] introduces a novel approach to enhance the reinforcement between binders and high-solid fillers in propellant formulations by grafting bonding groups onto the binder to form a neutral polymeric structure. For such a purpose, the authors employ a glycidyl azide polyol energetic thermoplastic elastomer binder with a –CN bonding group (GAP–ETPE), which was synthesized and tested with RDX-based model propellants. Furthermore, the mechanical analysis showed improved tensile strength (6.43 MPa) and strain (32.1%). Additionally, dynamic mechanical analysis indicated that increasing the RDX content raised the glass transition temperature (Tg) and the storage modulus. And finally, thermal analysis revealed four decomposition stages, enabling the authors to establish a thermal decomposition equation. These findings provide a promising method for improving mechanical properties and understanding thermal decomposition behavior, offering technical support for propellant combustion studies.

The study by Trindade and colleagues [20] explores the use of additive manufacturing, particularly fused deposition modeling, to produce customized orthoses that improve patient comfort and quality of life. For such purposes, a series of nine polymeric materials was evaluated through compressive, flexural, and tensile tests in both horizontal and vertical print orientations. The authors concluded that polycarbonate, polylactic acid, and ULTEM™ 1010 demonstrated superior mechanical properties and consistent performance across orientations, making them ideal for orthotic applications. Additionally, a finite element model of an ankle–foot orthosis was developed to simulate stress, strain, and deformation under static conditions. Results highlight ULTEM™ 1010 as the most durable and high-performing material, providing guidance for optimizing orthotic fabrication.

Costa and coworkers [21] examine the notch effect on semicrystalline PVDF using U- and V-notch geometries of varying depths, performing tensile tests at 23 °C and combining Digital Image Correlation (DIC) and Finite Element Analysis (FEA). Both unnotched and notched specimens were analyzed to compare global mechanical curves and local strain maps. Thus, the results showed that notch geometry and depth significantly reduce load and displacement compared to unnotched samples, with strain maps confirming localized strain concentration near notch tips. Additionally, FEA demonstrated strong agreement with experimental data globally and reasonable accuracy locally within 0.5 mm of the notch region. Overall, DIC and FEA proved effective for evaluating notch behavior in PVDF used as pressure sheaths.

The work by Abdellah et al. [22] focused on composite materials, valued for their high strength-to-weight ratio, which are widely used in the aerospace, automotive, and shipbuilding industries to reduce energy consumption. They pay attention to the damage behavior, which changes significantly when stress discontinuities, such as holes, are introduced into the composite. This study investigates the effect of multiple holes on carbon fiber composites using a progressive damage model and finite element analysis (FEA). In this way, two-hole configurations were analyzed along longitudinal and transverse directions relative to the load. Results show that additional holes can act as stress-relief features, reducing stress by up to 17% when aligned longitudinally. Thus, a cohesive zone model was applied to develop a simplified analytical method for predicting the nominal strength of multi-hole laminates based on unnotched plate properties. They conclude that the model closely matched experimental data, and design tables were provided to support material selection and structural optimization.

Mohammed, Abdellah and colleagues [23] investigate the prediction of mode I fracture energy in Graphite-reinforced composite laminates. These types of materials are widely used due to their versatility and high-performance properties, but have the problem that delamination remains the most critical failure mode. Thus, the subject of this study is to predict mode I interlaminar fracture energy using the Virtual Crack Closure Technique (VCCT) integrated with finite element modeling (FEM) on double cantilever beam (DCB) specimens. Additionally, the authors developed a simple analytical model based on material strength and stiffness to calculate the critical fracture energy. They conclude that both approaches showed strong agreement with experimental results, yielding an error margin of 5%, which confirms their accuracy and reliability for evaluating delamination behavior under service conditions.

The article by Morokov et al. [24] investigates the effect of adding single-walled carbon nanotubes (CNTs) to the polymer matrix of carbon fiber laminates to enhance strength and resistance to mechanical loads. In this context, the development of impact damage was analyzed using high-resolution ultrasound imaging for laminates with CNT concentrations ranging from 0 to 0.5 wt%. Further, their results show that CNT addition reduces damage in the upper and lower layers but increases damage in the middle plies. These findings were discussed alongside impact history data, providing insights into the role of CNTs in damage propagation within composite laminates.

The investigation by Zhang et al. [25] evaluates the mechanical properties of ABS parts produced via fused deposition modeling (FDM) using a combination of experimental and numerical methods. In this way, a series of ABS specimens underwent tensile testing on a universal testing machine, while finite element analysis (FEA) in ANSYS 2021 simulated stress and deformation behavior under varying conditions, including pre-stretching and temperature gradients. Experimental results showed a maximum tensile force of 7.3 kN, upper yield force of 3.7 kN, and lower yield force of 3.2 kN, indicating high strength and toughness. Additionally, non-proportional elongation reached 6%, and the performance enhancement factor compared to traditional manufacturing was 1.1, aligning with reinforced ABS standards. Finally, the FEA results validated experimental findings, confirming 15 mm of plastic deformation before fracture, consistent with ABS’s ductile nature.

Finally, the research by Amaro et al. [26] focused on soft biological tissues that exhibit highly nonlinear and anisotropic mechanical behavior, posing challenges for replicating these properties in engineered materials for biomedical applications such as surgical simulation and device testing. This study presents a framework for reproducing the nonlinear stress–strain response of soft tissue using 3D-printed models. For such purposes, two polymers—thermoplastic polyurethane (TPU) and thermoplastic elastomer (TPE)—were selected for their tunable hardness and elasticity. The authors conducted a parametric study to examine the effects of Shore A hardness (60 A–100 A), infill density (0–100%), and shell number (0–2) on tensile performance. Additionally, mechanical testing provided stress–strain curves to evaluate structural reliability and functional behavior. In conclusion, these findings demonstrate the potential of additive manufacturing for anatomical reproduction and for accurate replication of mechanical properties in soft tissue models.

In summary, as Guest Editors of this Special Issue, and in light of the contributions to this Special Issue, we can affirm that the topic “Mechanical Behavior of Polymeric Materials” constitutes a fundamental framework within Polymer Science and Technology, both currently and in the foreseeable future. Accordingly, a third edition on this topic, scheduled for publication in 2026 in *Polymers*, is underway and now open for submissions. We welcome quality contributions.
